# Detection of HCV Persistent Infections in the Dental Pulp: A Novel Approach for the Detection of Past and Ancient Infections

**DOI:** 10.1371/journal.pone.0165272

**Published:** 2016-10-26

**Authors:** Layla Gomes Siravenha, Leonardo Quintão Siravenha, Lucimar Di Paula Madeira, Aldemir B. Oliveira-Filho, Luiz Fernando Almeida Machado, Rosimar Neris Martins Feitosa, Antonio Carlos Rosário Vallinoto, Marluísa de Oliveira Guimarães Ishak, Ricardo Ishak

**Affiliations:** 1 Laboratório de Virologia, Instituto de Ciências Biológicas, Universidade Federal do Pará, Guamá, Belém, Pará, Brasil; 2 Instituto de Estudos Costeiros, Universidade Federal do Pará, Bragança, Pará, Brasil; Central University of Tamil Nadu, INDIA

## Abstract

The dental pulp is a sterile highly vascularized tissue and has been commonly used as a biological material to detect the genome of infectious agents that reach the dental tissue. Indeed, the pulp is also used to reveal past and ancient infections in the field of paleomicrobiology. The present study aimed to detect the presence of Hepatitis C virus (HCV) in a small community (approximately 400 inhabitants) in the Amazon region of Brazil (Nossa Senhora do Perpetuo Socorro, Vizeu, Para, Brazil) and standardize a technique for the detection of the virus in the dental pulp. Serum samples were collected from 48 patients whose teeth were clinically recommended for surgical extraction. The group comprised an equal number of males and females, mostly agriculture workers and housewives, respectively. The majority (64.6%) received less than one minimum wage and were ill educated (less than four years of school years). An enzyme immune assay was used to detect antibodies to HCV and the 9 (18.8%) positive samples were submitted to nucleic acid extraction in the blood (using the EXTRAzol) and the pulp (QIAamp DNA Micro Kit e kit RNeasy Plus Micro). The pulp was removed using a modified protocol without the use of liquid nitrogen. Nucleic acid was found in 8 of the dental pulp, but in 7 of the blood samples. Sequencing of one of the samples showed the presence of genotype 1. Conclusions: A novel simplified methodology for the extraction and amplification of HCV nucleic acid was successful to detect the presence of persistent infections of the virus within the dental pulp tissue. The protocol may be helpful to detect past and ancient infections and to better understand the natural history of HCV.

## Introduction

*Hepatitis C virus* (HCV), a member of the family *Flaviviridae*, genus *Hepacivirus* [1], is an important human infectious agent due to the large array of human pathologies associated to its infection which are worsened by the perspective of further increase as a public health threat for the following years ahead [2]. Approximately 170 million persons are thought to be persistently infected [3,4] and at least 80% will develop severe diseases including chronic hepatitis, cirrhosis or liver cancer [5].

In Brazil, the prevalence of HCV is around 1% to 2% in the general population, but it ranges from 0.9% to 2.4% in the North region of the country [6,7] and it reaches more than 30% among injecting drug users [8]. Seroprevalence to HCV in the Pacui Island, Para, a small village within Cameta municipality, was 8.8% and RNA persistence was detected among 62.5% of the seroreactive subjects [9].

Despite the common routes of parenteral transmission (blood transfusion, drug injection, hemodialysis, acupuncture and tatooing), risk factors in the Amazon region usually involves the use of illicit drugs, sharing of sharpen instruments, the lack of condom use during sexual relations and the presence of other family members also infected with HCV [9].

The pursue of past (during the lifetime of the host) and ancient (in *post mortem* specimens) infections have been particularly successful when looking within the dental pulp, a time-resistant organic material. The dental pulp lies within a cavity which is limited by the dentin and the cement at the apical foramen [10,11]. It is a highly specialized tissue which contains several different cell components including fibroblasts, odontoblasts and mesenquimal cells, as well as, a large array of blood vessels [12] which communicate with other parts of the human body and allows the arrival of infectious agents to the dental pulp [10,13–15].

Among the several examples of the infection of the dental pulp it is noteworthy the recovery of HIV-1 integrated DNA in the fibroblasts of the dental pulp of an AIDS patient [16] and the detection of the DNA of *Yersinia pestis* originated from the teeth of skeletons dated from the XVI and XVIII centuries [17]. Later, the presence of *Y*. *pestis* DNA found in the dental pulp of a child and two adults, confirmed its causative role during plague epidemics in the XIV century [18], as well as its absence in skeletons thought to have died during epidemics in several parts of Europe, ruled out the participation of the bacterium [19]. Experimental animals were successfully infected with *Coxiella burnetti* and the bacterium DNA was recovered in the dental pulp and the liver [20,21].

The present paper shows for the first time a standardized technique for the detection of HCV in the dental pulp and the recovery of the nucleic acid among chronically infected persons.

## Materials and Methods

### Group Examined and Sample Collection

The examined group included 48 persons who were given a thorough clinical examination by a certified dentist when visiting the outpatient dental office in the village of Nossa Senhora do Perpetuo Socorro, municipality of Vizeu, Northeast of the State of Para (1°43'00.1"S 46°38'25.8"W). When the clinical situation required surgical removal of any tooth, the patient was invited to join the study. In case of acceptance, the patient answered a questionnaire (for demographic, social and cultural information), and had a blood sample (5 mL) collected before surgical removal of the tooth. All patients were required to be older than 18 years, be a resident of the village, have a clinical indication for surgical tooth removal and the dental pulp had to be in good anatomical and microbiological conditions.

Blood was collected using sterile vacuum tubes containing EDTA, plasma was removed from the cell mass by centrifugation at 3,000 rpm and kept at -20°C before use. Dental surgical removal was performed by a certified dental professional following a protocol which included the use of sterile instruments, anesthetics, removal of the tooth with forceps and the excess of gum tissue. The tooth was cleaned with 70% ethanol and placed in Falcon tubes with sterile PBS, pH 7.2 and placed at -70°C.

### Ethics Statement

The study was approved by the Ethics Committee of the Nucleo de Medicina Tropical of the Universidade Federal do Para (#1000384). Following the Guidelines and Rules for Research Involving Humans (Resolution 196 of the National Council of Health, Ministry of Health) all patients were informed with a written document and those who agreed to participate in the study signed an informed consent form.

### Serological Analysis

Antibody detection to HCV was performed using an enzyme immune assay (BioKit, Barcelona, Spain) according to the manufacturer’s recommendations. Positive samples were submitted for the detection of nucleic acid.

### Removal of the Pulp

The dental pulp was removed with the aid of a sterile spherical diamond round bur (KG Sorensen, Cotia, Brasil) and carborundum disks, maintained under refrigeration with cold water. The pulp was removed using sterile Hedstroen files (Maillefer Dentsply, Switzerland) with different calibers (#15 to #40) and a cutting spoon. The dental crown was cut by the pulpar chamber when needed; spherical round bur helped the access to the pulp tissue. Nucleic acid extraction was performed after removal of the pulp, under refrigeration. The pulp was frozen (-70°C), weighed (no more than 5mg was used), treated with RNAlater and completely grounded

### RNA Extraction

Extraction of nucleic acid from cellular and non cellular samples of the blood was performed using the EXTRAzol (Nanogen,S.p.A, Italy), according to the manufacturer’s instructions.

Extraction of nucleic acid of the pulp was performed using RNeasy Plus Micro kit (Qiagen, Austin, Texas,USA) without the use of liquid nitrogen. The sample was weighed (less than 5 mg), frozen to -70°C, ground with a sterile cutting spoon, and without thawing added 350 μL of RLT plus buffer. The lysate was placed into a QIAsheredder spin column inside a 3mL tube and centrifuged for 2 minutes at 14,000 rpm. The supernatant was carefully transferred to the gDNA Eliminator spin column, which was placed in a 2mL tube and centrifuged for 30 seconds at 14,000 rpm. Ethanol, 70%, was added (350 μL) to the filtrate, homogenized and transfered to the RNeasy MinElute spin column which was placed into a 2mL tube and centrifuged for 15 seconds at 14,000 rpm. The filtrate was then discarded.

To the column it was added 700 μL of RW1 buffer and centrifuged for 15 seconds at 14,000 rpm and the filtrate was then discarded. RPE buffer (500 μL) was added to the column and centrifuged for 15 seconds at 14,000 rpm to wash the membrane of the column and the filtrate was discarded. Ethanol, 80%, was added (500 μL) to the column and centrifuged for 2 minutes at 14,000 rpm and the filtrate was discarded. The column was placed in a 2mL tube and centrifuged for 5 minutes at 14,000 rpm and the filtrate was discarded. The column was finally placed into a 1,5mL tube added 14 μL of RNAse free water directly in the center of the column membrane and centrifuged for 1 minute at 14,000 rpm for elution of the RNA.

### Reverse Transcription

cDNA was prepared with the High Capacity cDNA Reverse Transcription kit (Applied Biosystems, USA) following the manufacturer’s protocol. The product was stored at -20°C before use.

### Real Time PCR for the Detection of HCV

The amplification of the 5’UTR region consisted of a reaction with a volume of 25 μL with 8.75 μL of water, 12.5 μL of the mix and 0.75 μL of assays and 3 μL of cDNA. GAPDH was used as an internal control using the same protocol, excluding cDNA. The amplification reaction was performed in a StepOnePlus™ Real-Time PCR Systems (Life Technologies, USA), using the following protocol: one cycle of 50°C for 2 minutes, one cycle of 95°C for 10 minutes (for the initial denaturation), 45 cycles of 94°C for 15 seconds and 60°C for 1 minute, using the following primers: HCV_NCR5-C93F (5’- GCTCAATGCCTGGAGATTTGG -3’), HCV_NCR5-C93R (5’- CTTTCGCGACCCAACACTAC -3’) and probe HCV_NCR5-C93M2 (5’- [FAM]-TCGGCTAGCAGTCTCG -3’) were used as previously described [22] and for GAPDH (GAPDH-F 5’-GAAGGTGAAGGTCGGAGTC-3’; GAPDH-R 5’-GAAGATGGTGATGGGATTTC-3’; probe 5’-[FAM]-CAAGCTTCCCGTTCTCAGCC-[TAMRA] -3’). Data analysis was performed with the aid of the New StepOne™ Software v2.2.1.

### Sequencing and Phylogenetic Analysis

After molecular diagnosis of viral infection, positive samples were selected for amplification of the 5’UTR of HCV as previously described [22]. The product of the amplification was run on a 1.5% agarose gel stained with ethidium bromide and then visualized with ultraviolet light. The amplified fragment was sequenced in both directions according to Sanger *et al*. [23] using an ABI Prism 310 Genetic Analyzer and the commercial kit Big Dye Cycle Sequencing Standard (Life Technologies, USA).

Nucleotide sequences were edited and aligned using BioEdit software [24]. The final alignment was performed using Modelgenerator software [25] aiming to select, according to the corrected Akaike information criterion, the best model to apply for the phylogenetic analysis. These parameters were used in the PHYML program version 2.4.4 [26] to infer trees by the maximum-likelihood method. The statistical reliance of the tree topologies was evaluated using 1,000 bootstrap replicates.

The final phylogenetic tree was obtained by majority-rule consensus and then edited using graphic resources found in the FigTree software [27]. Nucleotide sequences obtained from the National Center of Biotechnology Information were added to the alignment and used to construct the phylogenetic tree. The nucleotide sequence obtained in this study was deposited in the National Center of Biotechnology (#KU955710).

## Results

The group investigated (n = 48), comprised approximately 12% of the inhabitants of the village, showed an equal proportion of males and females with a mean age of 36.1 years (age range 18–94) for males and 36.5 (age range 18–62) for females. The majority (64.6%) survived with less than one minimum wage. Both sexes referred their first sexual relation around 16 years old, 65.5% never used condoms and all were heterosexuals. One was a sexual partner of an injecting drug user and one was infected by HIV-1.

Most males (58.3%) worked in agriculture and 41.6% of the females were housewives; the number of school years was less than four among 45.9% and 33.3%, respectively. More than 90% of men and women were born in the State of Para and the majority was married (50% and 79.1%, respectively).

**Fig 1:**
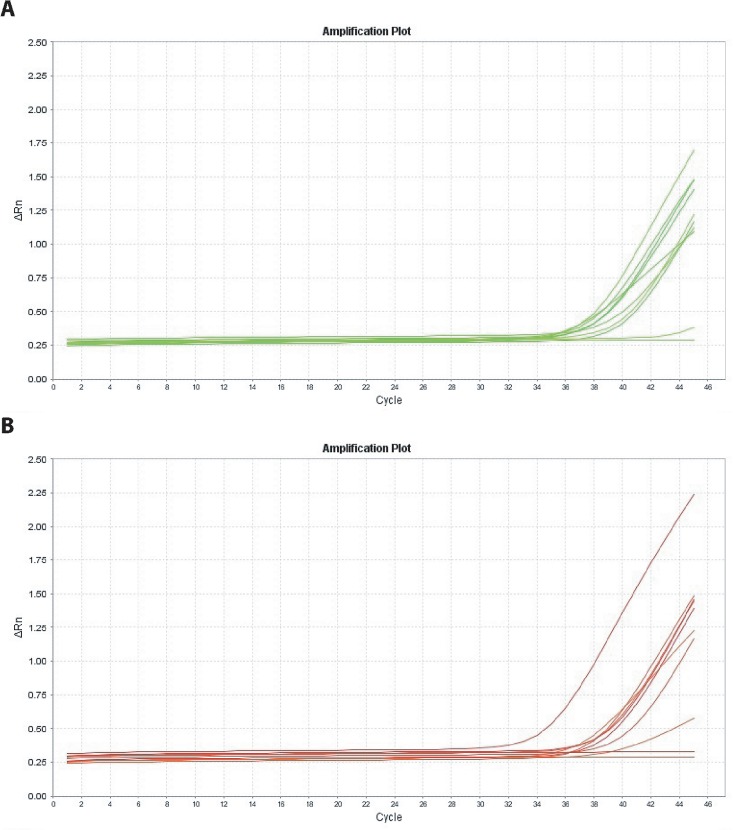
qPCR detection of HCV. (A) in the dental pulp and (B) in the blood. ΔRn (Delta Rn) is the normalization of Rn (fluorescence of the reporter dye divided by the fluorescence of a passive reference dye) obtained by subtracting the baseline (ΔRn = Rn-baseline)

**Fig 2:**
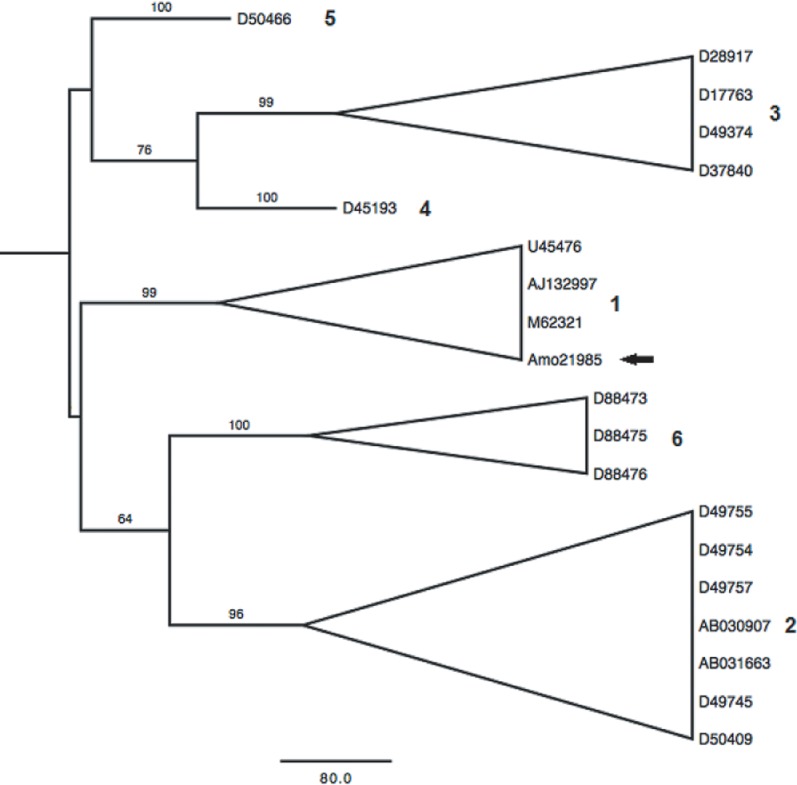
Phylogenetic analysis of the HCV isolated from the dental pulp. Maximum-likelihood phylogenetic tree derived from the alignment of 269 base pairs of the 5’UTR of hepatitis C virus detected in one of the positive individual.

The presence of antibodies to HCV was detected in 9 (18.8%) of the 48 individuals investigated. Nucleic acid of the virus was detected in 7 (77.8%) samples of the peripheral blood and in 8 (88.9%) samples of the dental pulp (Fig 1). Amplification of positive controls was present in all samples. One of the samples was sequenced and the phylogenetic tree showed it to belong to genotype 1 (Fig 2).

## Discussion

The present work aimed to standardize a methodology for removing the dental pulp for nucleic acid amplification in order to detect past and ancient HCV infections and to compare the positive findings of virus nucleic acid and the presence of antibodies to HCV.

The population group examined lived in a poor rural area in the Northeast of the State of Para, which survives basically in agriculture with less than one minimum wage. The population is clearly deprived of health care and access to medical services, to general health information, and sexual guidance directed to avoid infections not only by sexual and blood transmitted agents, but also to non infectious diseases.

Despite of any information they are able to obtain, it is generally difficult to understand either simple or complex data, as formal education (number of school years) is low, which is a common finding in most rural areas of the country, but is even worse in the Amazon region of Brazil. A clear indication is their sexual initiation early in life and the lack of condom use, an enhancing risk factor for the dissemination of infections.

It is a common procedure nowadays by dental practitioners the refusal of tooth removal, but the clinical situation of the patients examined urged immediate action. Among the most common reasons for tooth removal, included excrutiating tooth pain, signs of heavy bacterial infection and severe inflammation of the gums. Again, their understanding of simple information was in prejudice of their lack of formal education and further impaired by the absence of financial resources from their familial income to direct towards oral and dental welfare.

The presence of antibodies was detected in a high proportion of persons, contrary to what is generally seen in most parts of the world, in Brazil (1–2%) and in the Amazon (0.9–2.4%) region [6,7,28]. In a small community in the State of Para, in the municipality of Cameta, the prevalence of antibodies to HCV was found to be 8.8% and viral RNA was detected among 62.5% [9] of the positive samples which was still lower than in the present study. Sexual transmission of the virus could explain its large dissemination as it is a probable risk factor involved in the North region [29].

It is relevant to mention that the presence of nucleic acid was detected in most of the samples, whatever the origin of the biological material. Indeed, the dental pulp yielded one more sample than in the sera. To exclude the possibility of contamination, the sample was sequenced and further described as genotype 1, which is the most common genotype present in the North region of the country [30–32]. The reason for this is still not clearly explained, but higher replication rates in the pulp tissue cannot be discarded. Persistence of HCV is not commonly high among individuals with antibodies, and this finding was unusual. There is no other mention to such a high prevalence of virus persistence [33,34].

So far, the present study was the first to our knowledge to detect HCV nucleic acid from the dental pulp. The standardization of a simple methodology to remove the dental pulp was also successfully achieved. Some other approaches refer immediate removal of the pulp, following the tooth extraction. Glick et al. [35] refer tooth extraction of an HIV-1 patient and the immediate removal of the pulp for nuclei acid detection at the same day, and the finding of a high concentration of proviral DNA. Tran-Hung et al. [36] suggest to collect the pulp within 24 hours after dental extraction and used sterile resin for the removal. Mahmoudpour et al. [37] isolated *Enterococcus faecalis* from dead root canals of patients with or without symptoms. Removal of the pulp used an absorbent paper cone, which clearly causes a considerable loss of the pulp tissue, although this was not, apparently, a cause on impairment.

In the present study, as a consequence of the distance to the laboratory, the teeth were all placed in PBS for transportation and later (24 to 48 hours) frozen at -70°C. Apparently, there was no prejudice with this procedure for the amplification of HCV nucleic acid. Apparently, there was no external contamination and the loss of pulp tissue was reduced to a minimum (most of them weighed more than 5g). It is preferentially required that the operator has some knowledge of the tooth anatomy and to be skilled with the use of files and burs.

For the extraction of RNA the pulp was not frozen in liquid nitrogen before grinding. Freezing the sample (after weighing) at -70°C was sufficient to successfully obtain positive results during the procedure. This is a simplification of the method which provides a safer procedure, avoiding the use of a potential hazard.

The dental pulp is largely vascularized and therefore infectious agents present in the blood can easily reach the dental tissue both in humans and other animals [17,20,21,38]. The tissue has been used as a common source for the recovery of nucleic acid from past and ancient infections such as *Yersinia pestis* from the teeth of skeletons dating from the XVI and XVII centuries [17]. The entry of infectious agents into the tooth and, particularly, the dental pulp, seems to trigger the activation of several pathogen recognition receptors (PRR) in response to pathogen-associated molecular patterns (PAMP), as an important tissue devoted to the innate immune system [39].

The analysis of nucleic acid has been used to detect ancient molecules from different infectious agents which are used also to delineate historical patterns of infections and ancient migrations of human populations around the world. This new field of study, paleomicrobiology, started with the description of *Mycobacterium tuberculosis* DNA in an ancient skeleton and since then it helped to identify and characterize infectious agents and their geographical dissemination according to what is found in paleo specimens [40–42].

Description of some viruses has already been reported such as human papillomavirus and HTLV-1 in tissues dated from the IV and XV centuries respectively [43,44] and the influenza virus strain (H1N1) from 1918 in paraffin-embedded biopsies [45]. It is important to mention that these agents represent viruses which are maintained as a proviral genome and an RNA virus which shows a short replication cycle and an apparently short or absent viremia in the human host. It is important to point that HCV establishes a persistent infection in a small proportion of the infected persons, but without integration of its nucleic acid.

A novel simplified methodology for the extraction and amplification of nucleic acid of HCV from the dental pulp was standardized and the rate of success was higher than using serum, plasma or whole blood. The protocol was able to detect the presence of persistent infections of the virus within the dental pulp and is readly accessible; it may be used in a combined approach with archeology to detect past and ancient HCV infections in order to better understand the origin, emergence, evolution and dissemination history of HCV.
